# Aging Successfully Despite Limitations? Meanings and Perceptions of Aging Well Among Older Adults Living in Long-Term Care Institutions

**DOI:** 10.3390/geriatrics11020026

**Published:** 2026-02-28

**Authors:** Feliciano Villar, Nuria Ramón, Juan José Zacarés

**Affiliations:** 1 Department of Cognition, Development and Educational Psychology, Faculty of Psychology, University of Barcelona, 08035 Barcelona, Spain; nuriaramcor@gmail.com; 2 Department of Developmental and Educational Psychology, Faculty of Psychology, University of Valencia, 46021 Valencia, Spain; juan.j.zacares@uv.es

**Keywords:** aging well, successful aging, long-term care facilities, older adults, qualitative research, self-perceptions of aging, person-centered care

## Abstract

**Background/Objectives**: Dominant models of successful aging emphasize health, autonomy, and active engagement, often excluding older adults belonging to vulnerable groups, such as those living in long-term care facilities (LTCFs). This study aims to address this limitation by exploring how LTCF residents define “aging well” and by examining whether they perceive themselves as aging well according to their own criteria. **Methods**: A qualitative design was employed using semi-structured interviews with 30 residents aged 67–95 living in three long-term care facilities located in Barcelona, Spain. Interview transcripts were analyzed using inductive thematic analysis. **Results**: Five core themes emerged in the participants’ definitions of aging well: health, attitude, social ties, security, and activities. Health was the most frequently mentioned domain but was conceptualized in undemanding terms, focusing on basic autonomy and cognitive functioning. Psychological attitudes and meaningful social relationships were also key, alongside contextual factors, such as security and access to activities. Two-thirds of the participants perceived themselves as aging well, with justifications closely aligned with their personal definitions; negative self-perceptions were mainly associated with poor health, loss of autonomy, or loneliness. **Conclusions**: The findings suggest that, in contrast with academic definitions, LTCF residents define aging well in a broader, more context-sensitive manner, which allows them to view themselves positively despite their limitations. Person-centered care environments may play a crucial role in supporting aging well in institutional settings.

## 1. Introduction

Recent scholarly gerontological endeavors have increasingly promoted a more positive paradigm of aging, underscoring the potential for older adults to sustain high levels of physical health, psychological well-being, and social engagement. Central to this shift within gerontology is the concept of successful aging [1,2], which has significantly shaped both research agendas and policy initiatives regarding later life.

However, the dominant model of successful aging, despite its extensive adoption and impact, has faced considerable critique. Critics argue that its prescriptive criteria inadvertently establish an aspirational, yet exclusionary, standard for later life achievement [3,4]. This perspective often fails to account for the lived realities of a substantial portion of the older population, particularly those managing chronic disabilities, experiencing social vulnerability, or residing in institutional settings. The marginalization of these diverse experiences risks promoting a narrow, biased definition of “success”, thereby potentially exacerbating existing social inequities and contributing to the further social exclusion of vulnerable groups

The reason for this explanatory failure lies in the inherently divisive nature of the concept. According to Waddell et al. [5] (p. 4) “the term ‘successful aging’ is problematic due to the implied dichotomy of successful versus unsuccessful aging and, accordingly, precludes many from reaching “success” (e.g., those with disabilities)”. These authors propose ‘aging well’ as an alternative concept, as it may be more suitable for capturing the current understanding of optimal experience in late adulthood. In our study, without engaging in detailed conceptual definitions, we adopt the term ‘aging well’ as an operational and less constrained equivalent of the ideal of ‘successful aging’.

Although addressing conceptual limitations is important, empirical research investigating the meaning and the very possibility of aging well among these often-overlooked, vulnerable populations of older people would shed light on to how attain a more inclusive view of optimal ways of aging. However, such research remains limited thus far.

The present study aims to address this gap by examining how older adults residing in long-term care facilities (LTCFs) define aging well, by determining whether their views diverge from academic conceptualizations and from the lay perspectives of community-dwelling older adults, and by assessing the extent to which older adults living in LTCFs perceive themselves as aging well according to their own criteria.

### 1.1. How Successful Aging Has Been Understood?

Despite the lack of a universally accepted definition of successful aging [6,7,8], the seminal model proposed by Rowe and Kahn [1] has been the cornerstone of recent contemporary gerontological research in this area. They posited that successful aging achieved through the integration of three primary factors: (a) low probability of disease and disability, (b) high levels of cognitive and physical functional capacity, and (c) active participation in life, characterized by rich social connections and productive involvement. In addition, Rowe and Kahn assume that achieving a successful aging path fundamentally depends on lifestyles and, ultimately, on individual will. In their view, “successful aging is dependent upon individual choices and behaviors. It can be attained through individual choice and effort” [1] (p. 37). While this framework lends itself to promoting individual agency and designing interventions, it has been critiqued for underestimating the role of structural inequalities and societal context in shaping the aging experience [4]. In the last years, there has been an increasing conceptual and empirical effort to move beyond a ‘decontextualized’ understanding of successful aging. In this direction, Zhuo and Cao [9] propose incorporating four measures related to the environment into a revised model of successful aging: the inclusivity of disadvantaged groups, culture-specific adaptation, the balance between physical and social environments, and the dynamics of the entire life cycle.

Another critique leveled against the Rowe and Kahn model centers on its biomedical bias. Although the model includes both physical and social dimensions, the components are often interpreted and applied in a hierarchical fashion, with the physical dimension being the foundational tier of success in later life. In practice, the achievement of clinical and physiological standards, and specifically the absence of disability and the maintenance of high physical function, is often prioritized, and such components are the most frequently included within the many definitions of successful aging [7,10].

In response, alternative approaches have underscored psychological processes and adaptation as the core of positive ways of aging [11,12,13]. In these models, “success” is reframed as a dynamic process of adjusting to the inevitable resource diminution and biopsychosocial challenges associated with advancing age. The key outcome shifts from the compression of morbidity and delay to disability, which were central in Rowe and Kahn’s model, to the achievement of psychological well-being or happiness through effective coping and life management strategies [14].

Most academic approaches to successful aging tend to define it in middle-aged or youthful terms. They conceive “success” in later life as the preservation of the levels of biomedical or psychosocial functioning of earlier stages, and ignore older people’s experiences, which should be key to understanding and explaining aging. Some studies have attempted to fill this gap by exploring how older people define successful aging (e.g., [15,16,17,18,19,20,21]).

Results from systematic reviews [22,23,24] consistently demonstrate that older adults incorporate in their lay definitions a wider array of domains than typical academic models. While health and physical function remain present in lay definitions, their role is often less central than that of activity, frequently cited as the most important domain. Activity is broadly construed in lay models, encompassing not only physical exercise but also cognitive stimulation, social engagement, and meaningful relationships [16]. Even when physical activity appears, it is often contextualized by its connection to leisure and social pursuits rather than referring solely to functional ability [17]. Crucially, lay definitions heavily feature psychological factors, including optimism, self-acceptance, and well-being [18], alongside a strong emphasis on the capacity for acceptance and adjustment to loss and difficult circumstances [19,25].

Other less consistently reported but significant domains include spirituality and meaning in life [20] and the concept of a “good death”, aspects that could be particularly important among the oldest groups of older adults [22].

Finally, previous studies have also highlighted the importance of external resources in supporting individuals’ capacity to aging well, such as financial security [26] and environmental factors including access to high-quality social and health services [27]. This expanded multidimensionality in the lay criteria of successful aging has been confirmed through cross-cultural comparisons of older adults’ perspectives across diverse world regions [15,28]

### 1.2. Aging Well and Long-Term Care Institutions

A frequent critique to Rowe and Kahn’s model is that it sets unrealistic standards for achieving successful aging, standards that only a minority of privileged older people can attain [3,29]. As a consequence, such models make it extremely difficult, if not impossible, for most older people living in nursing homes to be classified as aging “successfully”.

Moving into a long-term care facility is typically accompanied with (or even determined by) the presence of highly and/or specialized care needs due to chronic illness or substantial functional impairments, including physical disability or cognitive decline. These very conditions are, by definition, the opposite of core criteria of the Rowe and Kahn model. Thus, the biomedical bias present in the model excludes older adults requiring institutional care due to health-related decline or lack of autonomy from aging successfully [30,31].

But even for residents able to maintain good functional autonomy and experiencing relatively good health, living in a long-term care facility may be a barrier to attain successful aging standards. Specifically, achieving the third criterion stated in Rowe and Kahn’s model, active engagement with life, is hardly possible in many long-term care institutions, since they present significant structural barriers to maintain close social ties, being involved in productive activities or participate in the life and decisions of the institution. The review by De Medeiros et al. [32] indirectly pointed to this issue by showing that institutionalization negatively affected the quality of life of older adults compared with those who were not institutionalized.

Evidence suggests that loneliness is a prevalent phenomenon for people living in long-term care homes [33,34,35], where opportunities to meet the need for intimacy, closeness, and touch are seriously restricted. Older adults who have either lost significant social relations, or were experiencing loneliness in the community, are more likely to move into a care home [36]. Opportunities for participation and other ways of civic engagement are also extremely limited for those living in a care home [37]. The literature highlights a number of barriers for promoting greater engagement, from those that are related to residents’ diminished personal resources (e.g., cognitive impairment) to practical issues (e.g., mobility, transportation) or contextual issues (e.g., staff attitudes towards residents’ participation, ageism, and ableism) [38,39].

However, if we move beyond the dominant Rowe and Kahn model to other academic models that emphasize psychological states (e.g., well-being or life satisfaction) and adaptative mechanism as the key for aging well [11,12], aspects that are also central in lay conceptions [18,28], older adults in long-term care facilities are more likely to be conceived as aging “successfully”. In the case of older adults living with disabilities, research suggests that elements such as adaptation and resilience (the capacity of coming to terms with limitations and making the most of one’s situation and available resources) or autonomy (the capacity to perform activities of daily living on their own and undertake activities that give pleasure and enjoyment) are particularly important in their lay definition of successful aging [40,41,42].

Despite there being no studies directly examining how older adults living in long-term care facilities define aging well, several investigations provide relevant insights. Guse and Masesar [43] identified four elements that residents considered essential for a good quality of life: —interaction with family and friends, personal qualities (e.g., feeling respected), “room and board” aspects (e.g., having a private room), and well-being factors (e.g., mobility or independence). Similarly, Cho et al. [44] reported five themes shaping daily life perceptions in nursing homes: enhanced comfort, aspirations to maintain physical and cognitive functioning, the desire for meaningful relationships, feelings of confinement and reduced autonomy, and acceptance and adaptation to institutional life. While not explicit definitions of successful aging, these experiences constitute the basis upon which residents may construct such understandings. Finally, Sunzi et al. [45] related aging well in a LTCF with residents’ everyday experience of dignity.

Defining aging well in more inclusive terms may facilitate one’s inclusion in such definition, which may be particularly important since studies suggest a strong relationship between a positive self-perception of aging and different quality of life indicators, including physical health, functioning, life satisfaction, self-esteem or self-efficacy [46,47,48]. In the case of collectives supposedly aging ‘unsuccessfully’ according to Rowe and Kahn’s definition, such as older people experiencing ill health or disability, it has been found that a wider definition lets them see themselves as aging well [20,43]. In the case of older people living in LTCFs, to the best of our knowledge, we still do not have studies focused on their lay definition of aging well or their self-perception of aging. However, some findings indicate that well-being and life satisfaction among residents in LTCFs may be higher than one exclusionary view of successful aging would suggest [49,50,51].

The aim of our study is to fill these gaps and explore (1) how older adults living in long-term care institutions define successful aging; and (2) determine to what extent older adults living in long-term care institutions perceived themselves as aging successfully according to their own definition.

## 2. Materials and Methods

### 2.1. Participants

Thirty people (18 women, 12 men) participated in this study. To participate, residents had to meet the following inclusion criteria: permanent residence in the LTCF for at least six months, age at least 65 and no diagnosis of mild cognitive impairment, dementia of any kind, or any other mental disorder. Participants’ ages ranged from 67 to 95 years old (M = 84.7 years). Eleven participants had completed primary education, 11 had some kind of secondary studies and three had university studies. Five participants reported not having completed any formal study. Table 1 provides a sociodemographic description of the participants.

Participants were recruited from three different LTCFs in the city of Barcelona, all of them under private management but included in the public network run by the Department of Social Services in Catalonia, Spain. To seek maximum variation, participating LTCFs were selected from different socio-economic neighborhoods of the city: one from a lower-income area (LTCF A), one from a higher-income area (LTCF B), and one from a middle-income area (LTCF C). All three facilities were relatively large, housing up to144 residents in LTCF A, up to 102 in LTCF B, and up to 160 in LTCF C.

### 2.2. Instrument

A semi-structured interview was designed and administered to all participants. The interview included questions regarding their understanding of aging well, their life priorities, future goals, and contributions to others. The structure of the interview partially followed the guidelines proposed by McAdams in his Life Story Interview Protocol [52]. For the present study, only the responses related to the meaning of aging well were analyzed (e.g., ‘What does aging well mean to you?’, ‘Do you know anyone in your situation who is aging well?’, ‘What characteristics lead you to think that this person is aging well?’, ‘In your case, do you feel you are aging well? Why?’)”. These questions were based on the ones included in previous qualitative studies on subjective views on aging well or successful aging [40,42].

### 2.3. Procedure

Author 1 presented the study to the management of each participating facility. Formal participation was established through a signed agreement outlining the research objectives and the standardized protocols for data collection. A designated liaison at each center compiled a comprehensive list of residents who met the established inclusion criteria. From these rosters, participants were randomly selected. We extracted the same number of participants from each center, ensuring a similar number of male and female in each one. No resident who was invited declined to participate in the study.

All interviews were conducted in a single session. Their duration ranged from 33 to 81 min, with an average length or 65 min. While the total duration of the interviews averaged over an hour, the specific responses pertinent to the final qualitative analysis were concentrated within a 10 to 15-min timeframe per participant. Interviews were conducted in private, quiet environments facilitated by LTCF management. To ensure data integrity, all sessions were audio-recorded.

The audio recordings were transcribed verbatim by Author 3 to ensure an accurate representation of the participants’ narratives. The research team engaged in a collaborative process of listening to, reviewing, and discussing the transcripts. By the 22nd and 23rd interviews, it was observed that participant responses increasingly mirrored previously collected data, with no novel concepts, themes, or nuances emerging, which may indicate that data saturation had been achieved [53]. However, the remaining scheduled interviews were conducted to reinforce the rigor and completeness of the dataset.

The study protocol received formal approval from the University of Barcelona Bioethics Committee (IRB 00003099) on 16 May 2022. Consistent with ethical best practices, all participants provided written informed consent prior to the commencement of data collection. The consent process involved a comprehensive disclosure of the study’s primary objectives and methodological approach, the specific procedures for data collection and management, and guarantees regarding anonymity and confidentiality. Participants were also explicitly informed of their voluntary participation, including the right to decline any specific question or to withdraw from the study at any point without penalty.

To protect participant identities, all data were pseudonymized prior to analysis; these pseudonyms were maintained throughout the presentation of the findings to ensure complete confidentiality.

### 2.4. Data Analysis

The qualitative data were analyzed thematically, adhering to the framework established by Braun and Clarke [54] and facilitated by ATLAS.ti 23 software. Adopting an inductive approach, the researchers derived codes directly from the raw data without the imposition of a predetermined conceptual framework.

The analysis proceeded through a systematic sequence: of four steps. Fist, we familiarized with data. Initial immersion involved iterative readings of the interview transcripts, complemented by the use of analytic memos in ATLAS.ti to document emerging insights. Second, units of meaning relevant to the research objectives were systematically coded. These codes were subsequently organized into preliminary themes and subthemes. Third, the internal coherence and external differentiation of the themes were critically reviewed. This phase involved defining the conceptual boundaries of each theme to establish a definitive thematic structure. In the final stage, the thematic structure was synthesized with the study’s objectives and situated within the context of the existing literature.

To ensure the credibility and trustworthiness of the findings, a collaborative analytic process was employed. Authors 1 and 2 performed the primary coding, followed by a peer-debriefing process [55] involving Author 3. During several consultative meetings, the peer-debriefer challenged both implicit and explicit assumptions. Any discrepancies in interpretation were discussed until a final consensus was reached, thereby strengthening the reliability of the thematic outcomes.

## 3. Results

### 3.1. The Meaning of Successful Aging

Concerning the first objective of our study, we identified five main themes in our interviewees’ responses in relation to the definition of successful aging: Health (including mentions referring the preservation of health, autonomy or cognitive abilities), Attitude (including mentions to personality traits or attitudes), Social Ties (including mentions to relationships with relatives or with staff), Security (including mentions to being physically or financially safe), and Activities (including mentions to scheduled and/or self-decided activities proving a sense of joy).

#### 3.1.1. Health

Health was the most frequently mentioned theme. Some of the responses mentioning health did so in a generic way (e.g., Juan José, a 96-year old man who said “aging well is to keep your health as much as possible”, I12), or identifying health simply with the absence of illness, (as Pedro, a 67 year-old man said: “It’s being healthy, totally healthy, that is, not suffering from any illness”, I20).

Other responses underscore some specific aspect of health, and particularly the preservation of cognitive abilities or at least the absence of severe deterioration of cognitive skills. Thys, Rafaela, an 85-year-old woman, told that aging well “Aging well is mostly about keeping your head together. As long as you’ve still got your wits about you, you’ve got plenty to be happy about”. Similarly, Remedios, an 87 year-old woman said that: “Keeping your head on straight so you can stay on top of things” (I01).

Finally, other responses associated aging well with the absence of any disabling condition. In these responses, the participant seemed to have a clear image of what is not ‘aging well’, related to dependency in daily living activities. For instance, Ana, an 87-year-old woman, rather than telling what aging well is, told what would be aging badly: “To me, aging well is being how I am right now. Getting sick and having to rely on everyone for everything… I know if I get sick it’ll have to happen, but that’s my idea of aging poorly” (I14). Similarly, Patrocinio, a 76-year-old man, identified lack of autonomy not just with aging well, but with a life worth living: “Listen, it’s all about staying healthy and being able to get by on your own. The day I need someone to bathe me and get me dressed, well, that’s I, you can just take me then” (I05).

#### 3.1.2. Attitude

The second most mentioned by them had to do with associating aging well with a certain attitude towards life.

In many cases, such attitudes were related to coming to terms with a less-than-ideal scenario, that is, nonetheless, unavoidable. Thus, Salvador, an 85-year-old man, said that “aging well means just taking it as it comes—you’re getting old and that’s that. You can’t dwell on it, because the years just keep adding up… but obviously, some people just can’t handle the reality of it” (I11).

In other instances, the participant identified maladaptive attitudes that impede or preclude the individual from achieving successful aging. This is exemplified in Elisa’s case, a 73-year-old woman, who seemed to assimilate aging well to avoiding a depressive or passive position towards life:

Aging well is about staying connected to the world. It’s about not letting yourself go… not just sitting in front of the TV all day with no drive to do anything. You need that spark, so you don’t fall into a hole. I know what depression is like; when you start seeing everything in a negative light, this physical and mental heaviness takes over. It ruins your life because it just paralyzes you, and you don’t want to move or see a soul.

(I21)

Expanding upon this notion, Ramon, a 91-year-old participant, explicitly articulated that successful aging is driven by personal volition and a ‘juvenile’ mindset. Her testimony links a successful aging process to an attitude of resilience in managing and even overcoming adversities (in her case, health-related challenges) that typically arise as one grows older, and supports that idea with a personal experience:

I think the secret is trying to keep that youthful spirit. You can’t turn back the clock, but with enough willpower, you can push yourself. The minute you give up, you’re lost. I was just at the physio… this foot of mine was operated on six years ago… and she told me that other people with much better legs than mine just say “I can’t’ go on” and ended up in a wheelchair. The doctors did their part, but I put in the work, too. And thanks to that, I’m still on my feet, even if I need this walker to do it.

(I30)

#### 3.1.3. Social Ties

The maintenance of social ties and social integration was also identified as a defining element of successful aging. In the majority of responses addressing this theme, the emphasis was placed on the quality and depth of relationships, rather than the quantity of these connections.

Specifically, familial bonds emerged as the most frequently cited, as illustrated by this response by Emiliano, a 75-year-old man:

Aging well means to have your kids and your family love you and be there for you like mine are…and to never have to worry about a thing. Thanks to my kids, I really have everything I need here, I can’t complain.

(I02)

Some responses emphasized that such family contact was not guaranteed when living in a nursing home, which underlines its importance. For instance, Tina, an 82-year-old woman, said that

The secret to growing old well is family affection. If they leave you out in the cold, you’re lost. It happens here every now and then…, sometimes it seems like the kids are uneasy about visiting the residence, so they end up sidelining their relatives.

(I18)

Other responses also highlight the importance of the staff and how they treat the residents and the relationships they establish with them. Fernando’s response, a 91-year-old man, illustrates that idea:

Aging well would be staying with my own people. Don’t get me wrong, it’s not bad here, but it’s not the same as home. No, they treat us well, and that’s great—the girls who look after us are really sweet. But if you were treated poorly, if it was mostly scorn or neglect, that’s not aging well. That would be a crying shame.

(I10)

#### 3.1.4. Security

Five participants emphasized in their responses that security was an important ingredient of successful aging. People mentioning security underlined the fact of having all of their needs satisfied, mostly basic everyday needs, so that life could be lived without strains, in a calm and peaceful way. This was the case of Leonor, a 95-year-old woman who identified aging well to be clean and well-fed: “aging well is get older just like I am here—at peace, kept clean, well-fed, and well-looked after” (I28).

Some responses placed a significant emphasis on financial stability as a core component of successful aging, viewing it as a primary source of security. Thus, Teófila, a 90-year-old woman, noted ‘To age well means having all needs met. Above all, it means having financial security and being free from economic concerns at this stage of life” (I07).

Mercedes, a 90 year-old woman, as well as relating security to financial resources, gave in her response the important role of children as providers and an additional source of security:

Aging well means wanting for nothing. It means when they organize a trip, you don’t have to worry about going because you’ve got the money. And I can afford it, I don’t have to stress. I’ve got a little bit of money tucked away; my kids keep it for me and manage it all. They are just so wonderful! They treat me so well. They tell me, ‘Mom, if you want to buy something, just say the word and I’ll get it for you. If there’s something you can’t get here, I’ll buy it.’ Just the other day, my daughter brought me a big bottle of some cheap cologne, but it’s just what I needed, you know? They wash us a lot here, today I just had a full shower and put some on for the first time, you see?

(I25)

#### 3.1.5. Activities

Finally, three respondents highlighted the engagement in leisure activities and the satisfaction derived from them as salient factors in achieving successful aging. For instance, Florencia, a 67-year-old woman, said that “Aging well means being able to do the things you love in your everyday life”. (I22)

Maria, an 85-year-old woman, also mentioned that the inclusion of scheduled activities in the care home facilitated a positive aging experience, identified as being busy and enjoying your daily life:

I really think staying busy does wonders. They put on so many activities here, so many people come by, everyone is doing their own thing, and there’s really something for everyone. We even asked for a bit more of the things we like, like classical music and the opera, and they’ve started doing those too, just to see what we enjoy. It makes the days go by just beautifully.

(I23)

### 3.2. Perceptions of Aging Successfully

As for results concerning the second objective, it is important to note that the results presented in the preceding section indicate that several participants conceptualized successful aging through the lens of their lived experiences. When prompted to define the construct, these individuals frequently engaged in self-positioning, perceiving themselves as aging well. This alignment was empirically supported by responses to the direct inquiry: “Do you think you are aging successfully?” Of the 30 participants, 20 (67%) provided an affirmative response.

The justifications provided for these self-assessments closely mirrored the thematic dimensions previously identified in the participants’ definitions of successful aging, suggesting a high degree of consistency between theoretical conceptualization and personal identity.

Among the participants who perceived themselves as aging well, the maintenance of health (and particularly cognitive health) and autonomy were particularly mentioned. For instance, Carmen, an 83-year-old woman, said:

Yes, I’d say so. For now, I’ve still got my wits about me, which is the main thing. So, yes, mostly because I can still take care of myself, like going to the bathroom and getting dressed on my own. Because, believe me, that’s not as easy as it looks.

(I23)

Some participants perceived themselves to be aging well by engaging in downward social comparison with fellow residents. This self-perception was particularly pronounced when comparing their own status to peers experiencing significant health declines, specifically those with cognitive impairments. This was the case of Hortensia’s response, a 90-year-old woman:

Oh yes, because I’m doing just fine. And really, there are others much worse off than me. There’s a whole floor here where the people are in a bad way—you can’t even have a proper word with them because they’ve started acting… well, a bit silly, you know?

(I26)

Beyond the dimensions of physical health and functional autonomy, other participants grounded their perception of aging well in the presence of specific psychological attributes. For instance, Emilia, an 87-year-old woman, identified certain personality traits as being fundamental to this self-perception: “I’m aging well because I’m so talkative and positive… I have an open personality, and I like to talk with people”. (I08)

Leonor, a 90-year-old woman, provided a more nuanced response by linking her perception of aging well to elements contingent upon personal agency. Consequently, successful aging is framed as an individual responsibility, reflecting a perceived internal locus of control and the capacity to exert influence over one’s environment and life events:

I’d say so, yes. I try my best to keep moving and stay active—that really matters a lot to me. And you’ve got to have something to look forward to. I’ve still got my little dreams; I don’t know how much longer they’ll last, but I’m living in the moment. I make sure my plants are growing nice and strong, and I’ve got a grandson getting married soon, which is just wonderful. I mean, you need a little something to be excited about, don’t you? Otherwise, what are we even doing here?

(I28)

Social ties, and particularly frequent relationships with relatives, also appeared as an important factor to perceive oneself as aging well. This is illustrated by Mateo’s response, a 75 year-old man: “Like I told you before, my kids just love me to pieces, and my grandson too. Everyone here is so fond of me, and it really makes me feel happy” (I02).

In some responses, the significance of social bonds extended beyond the provision of emotional well-being; these connections provide a sense of security and predictability in daily life. Participants identified this stability as a critical component in the self-perception of aging successfully. This is the case of Beatriz, an 81 year-old woman:

I’m aging just fine because I’ve got everything I need, and I’m very happy here. I have my dear friend who comes to visit, and she loves me a lot. My two sisters come by too. They can’t make it more often because they live quite a way away, but they’re here every Saturday and Sunday. We go out for a nice lunch and have a lovely time together. Truly, I’m doing very well right here.

(I16)

However, not all participants perceived themselves as aging well. Among the ten individuals who provided a negative assessment, their justifications were grounded in factors like those previously discussed. For instance, Claudia, an 88-year-old woman said that “I think not. I’m not aging well because my health has been a bit poor lately” (I24).

In other responses, the focus of the response shifted to a perceived decrease in autonomy, characterized by a loss of agency over one’s environment and the functional constraints that impede the realization of personal desires and activities. In this case, Patrocinio, a 76 year-old man gave an illustrative response, expressing frustration for the lack of independence:

I’m not aging well at all, because I have to have everything done for me. I’m just stuck here sitting in this chair all day long, that’s no way to grow old. If I could just have a walker, at least I could get myself down to the garden and not have everyone doing every little thing for me… I’ve just got no freedom anymore.

(I05)

Finally, some participants also expressed the absence of social contact, particularly with family members, where the frequency of interaction was lower than desired. This deficit was associated with feelings of loneliness, as exemplified in the account provided by Pilar, a 92-year-old woman, who noted the following:

No, I’m not doing so well. I’d have loved to grow old with my family around me. My son, he’s a wonderful boy, but he works such long hours, so I end up feeling lonely—truly lonely. He comes to see me every week, but the visit goes by in a heartbeat. The rest of the week just drags on and on… it feels like a whole month passes before I see him again.

(I19)

## 4. Discussion

Our study aimed to explore two main goals: (1) exploring how older adults living in long-term care institutions defined aging well; and (2) determining to what extent older adults living in long-term care institutions perceived themselves as aging well.

### 4.1. How Participants Define Aging Well

As for the first objective, health emerged as the most prominent theme, suggesting that this element is as central to the residents’ definitions of aging well as academic theories of successful aging propose [2]. However, the participants’ mentions of health refer to a fundamental, minimalist conception of the term, primarily linking it to the absence of severe illness, the maintenance of cognitive abilities, or autonomy understood as the capacity to perform basic activities of daily living (ADLs) independently. Such a diminished, less demanding view of health may facilitate individuals living in LTCFs in meeting aging-well criteria and perceiving themselves as aging well.

Nonetheless, beyond health-related mentions that align our findings with the biomedical perspective of the successful aging model (albeit in the diminished version argued above), other themes appear that are absent from Rowe and Kahn’s framework but present in studies on lay definitions of aging well. Among these, psychological and social factors are particularly noteworthy.

Regarding psychological aspects, participants highlighted the capacity to maintain a youthful and optimistic spirit, an outlook on life that enables them to overcome adversity and accept inevitable challenges without succumbing to distress. These factors, which appear in lay definitions provided by both healthy older adults [18] and those with disabilities [42], allow individuals to maintain their sense of agency and continue exercising control over their own aging process, transcending strictly biological and health-related constraints.

The significance of social ties and activities, also present in Rowe and Kahn’s successful aging model (although as a subsidiary factor) and in lay perspectives on aging well [16,40] is also evident in the participants’ responses. For these individuals, regular contact with family members and bonds with professional caregivers are essential for daily well-being and, consequently, form an integral part of their notion of aging well. However, the way social ties are conceptualized in their responses points to an element missing from academic definitions of successful aging and from most lay definitions: the critical role of context. Specifically, in this case, the conditions of the residential facility act as either facilitators of or barriers to aging well.

This relevance of the residential context is manifest in at least three themes: social ties, security, and activities. Regarding social ties, beyond family, the relationship with professionals and receiving respectful treatment from staff is fundamental, as these are the individuals with whom daily life is shared and upon whom the fulfillment of the residents’ needs largely depends. Similarly, some participants value a sense of security linked to having basic needs met (e.g., being clean and well-fed), which is also contingent upon the institution. Finally, the institution is viewed as a context responsible for providing stimulating social and leisure activities for the older adults residing there. While the importance of context also appears in definitions of aging well among people with disabilities [40,42] in relation to technical aids and accessibility, in the case of LTCFs, it refers to a much more restrictive environment, concerning the very nature and organization of relationships and activities within the institution.

Taken together, the definition of aging well emerging from these responses emphasizes, in addition to minimum health conditions, the maintenance of agency within a context that is, by definition, restricted. This context must provide certain conditions (dignified treatment, security, and stimulating activities) that lie beyond the resident’s direct control but have a decisive impact on their well-being and on preserving agency in those areas in which it is still possible. This simultaneous consideration of agency and context as fundamental elements of aging well has been highlighted by authors such as Wahl [56] as a necessary condition for a more inclusive vision of aging, one that can account for the experiences of diverse groups, including those living in residential care.

### 4.2. Perception of Oneself as Aging Well

Such a view on aging well clearly influences the perception of oneself as aging well, which was the second objective of the study. Most participants perceived themselves as aging well, which is facilitated by the definition of what it means to be “healthy” (just considering very basic criteria such as the absence of cognitive impairments), by valuing the fact of being able to express agency, by self-attributing psychological attitudes and traits (e.g., optimism), or by identifying positive qualities in the context in which they live (e.g., security, range of available activities, etc.).

Beyond its impact on self-esteem and subjective well-being, adopting a definition of aging well that is sufficiently broad, allowing individuals to self-identify as aging well even amidst limiting conditions, may be a catalyst for healthy behaviors and have positive implications for social life, protecting from loneliness and depression. Such a relationship between self-perceptions of aging and positive functioning across the biomedical, psychological, and social dimension, which has been proven among healthy and community-dwelling older adults [46], deserves more research attention in the case of older people living in LTCFs.

However, results also indicate that not every participant perceived themselves as aging well: one third of our participants did not. Examining their responses, it seems that the way older people living in LTCFs define aging well may promote perceiving themselves as successful agers, but at the same time is also risky, as many of the identified dimensions were out of one’s control. Health is one of such non-controllable dimensions, but we also found other elements, such as the frequency of contact with relatives, the dignified treatment by facility staff, the availability and accessibility of leisure activities, or the security provided by the environment. This lack of control underscores resident inherent vulnerability and their dependence on both others and an optimized environment.

### 4.3. Limitations and Contribution to the Field

The interpretation of these results is subject to certain constraints. Specifically, the small sample size, also restricted to a few institutions in the same country participating in the study, may limit the generalizability of the findings to the wider population of older adult residents in aged-care settings. Furthermore, the voluntary nature of participation must be acknowledged, as it introduces a potential self-selection bias. Individuals lacking the motivation to participate, or those precluded by physical infirmity or cognitive impairment, were unable to contribute their perspectives. Consequently, the findings are reflective of a specific resident profile, those both motivated and capable of engagement, and there is no assurance that the views of non-participants would align with the reported data.

Despite its limitations, our findings contribute to achieving a deeper understanding of what aging well is, a view that must consider the perspectives of older adults living in LTCFs. Our results suggest that academic approaches (and particularly, models of successful aging) may be too restrictive and exclusionary, not reflecting the experience of older adults living in LTCFs. Unlike many academic definitions, their definitions include a flexible and reduced view of health. In their view, psychological (e.g., agency, adaptation and having a positive attitude towards life) and contextual factors (e.g., security or social relationships) are at least as important as biomedical aspects. That view of aging well allows for considering themselves as meeting its criteria, supporting a positive view of oneself that may have important consequences for physical health and well-being.

### 4.4. Practical Implications

This study also has some practical implications. Since aging well seems to largely depend, in the case of older people living in LTCFs, on contextual elements out of their direct control, perhaps the most obvious is the crucial role of LTCFs in the provision of optimal conditions for promoting (or not) aging well among people living there. A culture of care that reinforces and gives opportunities for guaranteeing the resident’s agency, that pays attention to the relations and bonds between residents and committed staff, or that offers a range of activities and experiences that make life worth living is fundamental to also extend aging well among people living in institutions. It seems a culture of care far more in tune with person-centered care models than with traditional, task-oriented technocratic models of running an LTCF [39].

Elements such as higher staff capacity and home-like environments that support autonomy and social integration are linked to better functional outcomes and greater subjective well-being among residents [57]. Consequently, the presence of these organizational characteristics in long-term care facilities constitutes an essential contextual foundation for enabling the experience of aging well.

## Figures and Tables

**Table 1:** Sociodemographic characteristics of the participants in the study.

Code	Name	Gender	Age	Education Level	LTCF	Years Since Admission
I01	Remedios	F	87	Primary	A	<1
I02	Mateo	M	75	Secondary	A	2
I03	Elena	F	84	No formal studies	A	1
I04	Ángel	M	76	Secondary	A	2
I05	Patrocinio	M	76	Primary	A	3
I06	Raquel	F	93	No formal studies	A	1
I07	Teófila	F	90	Primary	A	2
I08	Emilia	F	87	Primary	A	3
I09	Rafaela	F	85	Secondary	A	2
I10	Fernando	M	91	Primary	A	2
I11	Salvador	M	85	Secondary	B	2
I12	Vicente	M	96	University	B	1
I13	Alberto	M	81	No formal studies	B	1
I14	Ana	F	87	Secondary	B	1
I15	Benjamín	M	89	Secondary	B	1
I16	Beatriz	F	81	Primary	B	1
I17	Carmen	F	88	Secondary	B	<1
I18	Agustina	F	82	University	B	2
I19	Pilar	F	92	Primary	B	4
I20	Pedro	M	67	Secondary	B	2
I21	Elisa	F	73	Secondary	C	3
I22	Florencia	F	67	Primary	C	5
I23	María	F	85	University	C	2
I24	Claudia	F	88	Secondary	C	<1
I25	Mercedes	F	90	No formal studies	C	<1
I26	Hortensia	F	90	No formal studies	C	4
I27	Francisco	M	81	Primary	C	2
I28	Leonor	F	95	Primary	C	2
I29	Javier	M	89	Primary	C	3
I30	Ramón	M	91	Secondary	C	1

## Data Availability

The data presented in this study are available on request from the corresponding author. Data are available on request.
